# Development of a community’s self-efficacy scale for preventing social isolation among community-dwelling older people (*Mimamori* Scale)

**DOI:** 10.1186/s12889-016-3857-4

**Published:** 2016-11-28

**Authors:** Etsuko Tadaka, Ayumi Kono, Eriko Ito, Yukiko Kanaya, Yuka Dai, Yuki Imamatsu, Waka Itoi

**Affiliations:** 1 Department of Community Health Nursing, Faculty of Medicine, Yokohama City University, 3-9 Fukuura, Kanazawa-ku, Yokohama, Kanagawa 236-0004 Japan; 2 Department of Home Health Care Nursing, School of Nursing, Osaka City University, 1-5-17 Asahi, Abeno-ku, Osaka, 545-0051 Japan; 3 Kamakura Women’s University, 6-1-3 Ōfuna, Kamakura, Kanagawa 247-0056 Japan; 4 Soka University, 1-236 Tangi-machi, Hachioji, Tokyo, 192-8577 Japan; 5 Teikyo University of Science, 2-2-1 Senjusakuragi, Adachi-ku, Tokyo, 120-0045 Japan

**Keywords:** Elderly, Social isolation, Community networking, Neighborhood, Scale development, Self-efficacy

## Abstract

**Background:**

Among older people in developed countries, social isolation leading to solitary death has become a public health issue of vital importance. Such isolation could be prevented by monitoring at-risk individuals at the neighborhood level and by implementing supportive networks at the community level. However, a means of measuring community confidence in these measures has not been established. This study is aimed at developing the Community’s Self-Efficacy Scale (CSES; *Mimamori* scale in Japanese) for community members preventing social isolation among older people.

**Methods:**

The CSES is a self-administered questionnaire developed on the basis of Bandura’s self-efficacy theory. The survey was given to a general population (GEN) sample (*n* = 6,000) and community volunteer (CVOL) sample (*n* = 1,297). Construct validity was determined using confirmatory factor analysis. Internal consistency was calculated using Cronbach’s alpha. The Generative Concern Scale (GCS-R) and Brief Sense of Community Scale (BSCS) were also administered to assess criterion-related validity of the CSES.

**Results:**

In total, 3,484 and 859 valid responses were received in the GEN and CVOL groups, respectively. The confirmatory factor analysis identified eight items from two domains—community network and neighborhood watch—with goodness of fit index = 0.984, adjusted goodness of fit index = 0.970, comparative fit index = 0.988, and root mean square error of approximation = 0.047. Cronbach’s alpha for the entire CSES was 0.87 and for the subscales was 0.80 and higher. The score of the entire CSES was positively correlated with the GCS-R in both the GEN (*r* = 0.80, *p* < 0.001) and CVOL (*r* = 0.86, *p* < 0.001) samples.

**Conclusions:**

The CSES demonstrated adequate reliability and validity for assessing a community’s self-efficacy to aid in its preventing social isolation among older people. The scale is potentially useful for promoting health policies, practices, and interventions within communities. This may help prevent social isolation among older people and contribute to overall well-being in aging societies in Japan and abroad.

**Electronic supplementary material:**

The online version of this article (doi:10.1186/s12889-016-3857-4) contains supplementary material, which is available to authorized users.

## Background

The current unprecedented rise in solitary living is one of the most significant social changes of the modern world. Individuals living alone now account for more than one-third of all households in France, Germany, and the United Kingdom, and more than one-quarter of all households in the United States, Canada, and Japan [1]. Japan in particular has witnessed rapidly and dramatically transforming living arrangements of the nation’s older people. In 2015, 26.3% of Japan’s older population was living alone, up from 13.1% in 1985; this change has occurred in conjunction with an unprecedented aging of the population. Individuals <65 years old comprised 26.0% of the Japanese population, the highest percentage in the world, as of 2016 [2], and this figure is estimated to reach 30.3% by 2025 and 40.5% by 2055 [3]. At the same time, close community relationships are diminishing in Japan. The Cabinet Office’s National Census showed that approximately 30% of Japan’s residents believe that community ties have weakened over the past decade, and that this declining sense of community has worsened public safety [4]. These rapid and dramatic changes in Japanese society have led to several public health problems, including the social isolation of older people.

Although the concept of social isolation has been defined in various ways in the literature [5–8], many authors describe it as lack of a social network. This notion includes personal contact with others and deficient social support, such as emotional, informational, and instrumental support (i.e., offering assistance). Older people are at the highest risk of social isolation, and social isolation has been identified as a risk factor for poor health [9], reduced well-being [10], and increased mortality [11]. In particular, social isolation is associated with solitary death, in which an individual dies at home alone and unnoticed by others. In such cases, the body of the deceased may be left unattended for days, months, or even years. It is estimated that nearly 30,000 older people die such deaths each year in Japan [12]. Greater attention has recently been directed toward averting this tragic outcome. A systematic review of intervention studies published in English before 2009 [5] showed that group-based or individual interventions offering social activities or support groups among socially isolated older people improved at least one positive outcome, such as decreased loneliness [13], increased perceived social support [14], improved cognition [15], or decreased mortality [16]. A systematic review of interventions published in 2009–2013 [17] showed that only one intervention [18]—group-based reminiscence therapy—was successful in reducing both social isolation and depression in older people. However, these interventions only targeted socially isolated older people themselves, and not the community as a whole.

Thirty percent of older Japanese individuals who live alone do not regularly communicate with their neighbors [4]. Socially isolated older people tend to have less communication with their neighbors and are less likely to seek help from others. The key to preventing social isolation may lie not only in older people themselves, but also in the communities in which they live. Traditionally, communities have been a primary source of public well-being, and many studies have demonstrated their role in the health of older people [19–28]. Supportive relationships within a community can positively affect social participation and social capital for older people [26]. Conversely, poor relationships can pose difficulties for obtaining support, especially for older people living alone [27]. Fukukawa [29] demonstrated that the most helpful individuals for preventing social isolation and solitary death were not family members in distant locations, but nearby neighbors. Therefore, the roots of social isolation leading to solitary death in present-day communities may lie in the weakening of the community’s *mimamori*, as it is known in Japan, for older neighbors. *Mimamori* combines the two Japanese words: m*i*, meaning “watch” which derives from eyes, and *mamori*, meaning “guard” which derives from hands. *Mimamori* is particularly relevant for two major domains: interpersonal relationships and support networks. Social isolation is potentially preventable by watching over one another at the neighborhood level and implementing supportive networks at the closer community level. However, as of yet, there is no scale to measure the strength of such a community resource.

This paper discusses development of a Community’s Self-Efficacy scale for preventing social isolation among older people by two groups: the general population (hereinafter “GEN”) and community volunteers (hereinafter “CVOL”). According to Bandura, perceived efficacy reflects beliefs about one’s capacity for specific achievements, given domain-specific obstacles [30]. The scale developed herein is based on self-efficacy for two reasons. First, Bandura’s original work suggests that it correlates strongly with key aspects of performance, including goal-setting, hard work, and improved learning and achievement [30]. Second, the community’s beliefs about its capacity for achievement are a primary indicator of its potential ability to prevent social isolation of older people. Members of the general population are expected to be the primary resource for prevention, while community volunteers are expected to promote prevention in a variety of community health networks and organizations [31].

This study aims to develop a framework for the Community’s Self-Efficacy Scale for preventing social isolation among older people (CSES; *Mimamori Scale* in Japanese) and to present findings on the psychometric properties of the scale. The overarching goal is to promote well-being both among older people and the community as a whole.

## Methods

### Phase 1: Developing the instrument

First, a research team developed and reviewed a pool of items. This pool was based on literature reviews [19–27, 30, 32–36] and qualitative data from our previous surveys of public health experts, community members, and older people living alone [37, 38]. Criteria for inclusion of an item were based on three viewpoints: the degree to which the given item reflected the definition of preventing social isolation of community-dwelling older people; the clarity of logic, meaning, and readability of the given item for the GEN and CVOL groups; and the practical usefulness of items for those two groups.

Next, the pool of items was reviewed by eight experts, including community health nurses and social workers, to assess its validity, readability, and practical usefulness for the GEN and CVOL groups. Consequently, the initial CSES was refined to 24 items, consisting of three preliminary dimensions—knowledge, attitude, and behavior—that were focused on community-dwelling older individuals (eight items), neighborhood (eight items), and community (eight items). A four-point Likert-type scale was used (0 = not confident at all, 1 = slightly unconfident, 2 = slightly confident, 3 = completely confident), with the higher scores thus indicating higher self-efficacy.

A pilot study was then administered for applying item analysis and exploratory factor analysis to investigate the reliability and convergent validity of the initial CSES. The self-administered questionnaire was conducted in a convenience cohort of 297 community-dwelling adults recruited from community support centers in two cities in Japan. The mean age of the cohort was 67.2 (standard deviation [SD]: 7.3) years; with 163 (54.8%) women and 134 (45.2%) men. The criteria for item analysis included the rates of response difficulty (missing data: <5.0%), distribution (absolute values of skewness and kurtosis <1.0), and item-to-total correlation (>0.7).

The pilot study showed that 12 items were omitted and 12 retained from the 24 items of the initial CSES. Exploratory factor analysis with promax rotation was conducted on the 12 retained items. The latent root criterion (>1.0) and scree plot suggested a two-factor model (63.35% of the total observed variance); orthogonal (varimax) rotation generated comparable results. The first factor (seven items) was tentatively interpreted as “neighborhood watch” and the second (five items) as “community network.” In these two factors of 12 items, the cumulative contribution was 52.2% and Cronbach’s alpha was 0.86.

### Phase 2: Validating the instrument

#### Sample

The principal survey involved a GEN sample (*n* = 6,000) and CVOL sample (*n* = 1,297) in two major cities in Japan. The GEN sample was randomly obtained via the National Basic Resident Registration System administered by the Ministry of Internal Affairs and Communications of Japan. The self-administered questionnaire was sent by postal mail to 6,000 residents aged ≥55 years, using nationally representative quotas for age and sex. Of these, 3,605 (60.1%) responded and 3,484 (58.1%) questionnaires with valid responses were available for analysis.

The CVOL sample was openly recruited at community meetings administered by the local government. CVOLs were residents found to have carried out a wide range of activities for the sake of community welfare, commissioned by the Ministry of Health, Labour and Welfare of Japan. The same self-administered questionnaire was sent to 1,297 CVOLs by postal mail. Of these, 894 (68.9%) responded and 859 (66.2%) questionnaires with valid responses were available for analysis. Table 1 gives a summary of the study subjects.

**Table 1:** Study response flow

Population	GEN	CVOL
Registered	*n* = 6000 (100.0%)	*n* = 1297 (100.0%)
Responded	*n* = 3605 (60.1%)	*n* = 894 (68.9%)
Valid	*n* = 3484 (58.1%)	*n* = 859 (66.2%)

### Measures

Age, sex, living arrangement, marital status, years of living in the area, and employment were collected as demographic data items.

Two measures were used to assess the validity of the CSES. One measure was the revised Generative Concern Scale (GCS-R) [39], a Japanese version of the Loyola Generativity Scale [40]. “Generativity” is a term originally coined by Erik Erikson in 1950 to denote a concern and motivation for formulating and guiding the next generation and society as a whole. The GCS-R includes 20 items, scored from 0 (strongly disagree) to 4 (strongly agree), providing a range of 0–80. High scores on the GCS-R indicate a high level of generativity. Cronbach’s alpha of the GCS-R was 0.90 in the present study.

Another measure was the Brief Sense of Community Scale (BSCS), based on the theory of McMillan and Chavis [41]. Central concepts of the sense of community are the feelings members have of belonging, feelings that members matter to one another and the group, and a shared faith that members’ needs will be met through commitment to togetherness in a community. The BSCS includes eight items scored from 0 (strongly disagree) to 4 (strongly agree), providing a range of 0–32. High BSCS scores indicate a high level of sense of community. Because the BSCS had never been used in Japan, we translated it using a trained bilingual translator. Cronbach’s alpha of the BSCS was 0.90 in this study.

### Statistical analysis

IBM SPSS Amos 20 (SPSS Inc., Chicago, IL, USA) and SAS version 9.2 statistical software were used to perform all statistical analyses.

Exploratory factor analysis with promax rotation was performed on the initial, Phase 1, version of the CSES in the two sample groups. The optimal number of factors was determined by sequentially using latent root criteria (eigenvalues >1.0) and a scree plot. Item loadings needed to exceed 0.40. Factor reliability was determined using Cronbach’s alpha ≥0.70 and item-total correlations ≥0.70. Confirmatory factor analysis (CFA) was then performed using LISREL on the confirmed, Phase 2, version of the CSES in the two sample groups. Goodness-of-fit index (GFI), adjusted goodness-of-fit index (AGFI), comparative fit index (CFI), and root-mean-square error of approximation (RMSEA) were used to evaluate the data model fit. The model was accepted if the GFI, AGFI, and CFI indices were ≥0.90 and the RMSEA was ≤0.05. Correlational analysis was used to evaluate the criterion-related validity of the confirmed version of the CSES on the GCS-R and BSCS indices, and a correlation of ≥0.70 was considered adequate. Cronbach’s alpha was used to evaluate the internal consistency of the confirmed version of the CSES, with a value of ≥0.7 considered adequate.

## Results

### Respondent characteristics

Table 2 shows respondent characteristics. There were 3,484 valid responses in the GEN sample and 859 in the CVOL sample. In the GEN group, 51.7% of the study subjects were female and 71.0% were ≥65 years. In the CVOL group, 60.9% of the study subjects were female and 65.0% were ≥65 years.

**Table 2:** Respondent characteristics

	*N* (%)	
	GEN	CVOL
	*N* = 3484 (100.0)	*N* = 859 (100.0)
Sex		
Female	1800 (51.7)	523 (60.9)
Age		
<55 55–60 60–65 65–70 70–75 75–80 80–85 85–90 90–95 >95	- 419 (12.0) 590 (16.9) 829 (23.8) 716 (20.6) 524 (15.0) 276 (7.9) 95 (2.7) 32 (0.9) 3 (0.1)	70 (8.2) 75 (8.7) 157 (18.3) 236 (27.5) 241 (28.1) 59 (6.9) 17 (2.0) 4 (0.5) - -
Living arrangement		
Live alone Couple Live with children Other	431 (12.6) 1451 (42.3) 1430 (41.7) 122 (3.6)	75 (8.8) 369 (43.1) 394 (46.0) 18 (2.1)
Years living in the city		
<10 10–20 20–20 >30	393 (11.3) 540 (15.6) 721 (20.8) 1816 (52.3)	22 (2.6) 29 (3.4) 66 (7.7) 742 (86.4)
Born in the city		
Yes	915 (26.4)	226 (26.4)
Employed		
Yes	1199 (35.0)	232 (27.2)

### Factor structure

**Fig. 1:**
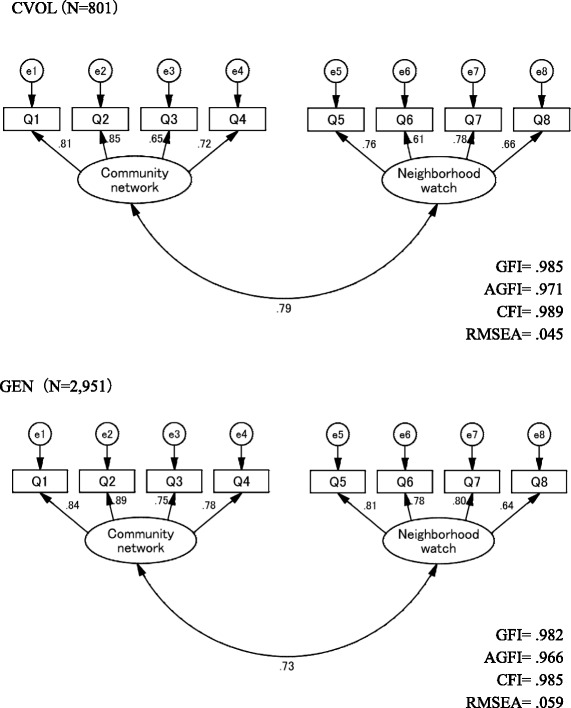
CSES confirmatory factor analysis

Exploratory factor analysis with promax rotation was performed on the eight remaining items of the initial version of the CSES in the GEN and CVOL samples. The latent root criterion suggested a two-factor model; inspection of the scree plot showed a noticeable difference in slope after the first two eigenvalues in both the GEN and CVOL samples. Orthogonal (varimax) rotation produced comparable results. In our interpretation, factor I included four items (Q1–Q4) interpretable as “self-efficacy of community network”, building a community network and community.” Factor II included four items (Q5–Q8) interpretable as “self-efficacy of neighborhood watch”, keeping watch over older people in the neighborhood and neighbors safety and well-being (Table 3). These two factors—community network and neighborhood watch—were entered as two latent factors in a CFA model. All fit indices indicate a good data model fit in the CVOL sample (GFI = 0.985; AGFI = 0.971; CFI = 0.989; RMSEA = 0.045) and GEN sample (GFI = 0.982; AGFI = 0.966; CFI = 0.985; RMSEA = 0.059) (Fig. 1).

**Table 3:** CSES exploratory factor analysis

		GEN *N* = 3,484			CVOL *N* = 859		
		Factor I	Factor II	Communality	Factor I	Factor II	Communality
Community network							
Q1	I can participate in the activities or volunteer work of my neighborhood association.	0.86	0.01	0.73	0.80	0.03	0.66
Q2	I can create an environment in which my neighbors can comfortably gather.	0.80	0.11	0.75	0.76	0.10	0.69
Q3	I can encourage nearby neighbors to come out to gatherings.	0.76	0.17	0.59	0.72	0.03	0.48
Q4	I can discuss my concerns about residents at neighborhood gatherings or community meetings held by local government.	0.68	0.15	0.62	0.62	0.16	0.54
Neighborhood watch							
Q5	I can check in on elderly neighbors if I do not see them for a few days.	0.05	0.78	0.64	0.04	0.75	0.59
Q6	I can help older neighbors with grocery shopping, garbage disposal, and other chores.	0.05	0.77	0.60	0.04	0.68	0.43
Q7	I can check in on neighborhood households where there are no signs of activity there.	0.08	0.73	0.65	0.18	0.63	0.58
Q8	When I notice a person I do not know in the neighborhood, I can speak to them.	0.07	0.62	0.45	0.14	0.65	0.45
Contribution %		0.24	0.28	0.52	0.32	0.22	0.54
Cumulative contribution %		0.24	0.52	0.52	0.32	0.54	0.54

### Internal consistency and validity

Mean scores on the final CSES were 10.2 (SD: 5.2) in the GEN sample and 13.8 (SD: 4.6) in the CVOL sample, with values of skewness and kurtosis of within ± 1. The CVOL group had a significantly higher score than the GEN group in all CSES versions (*p* < 0.001). Cronbach’s alpha for the CSES, “community network” (Factor I) and “neighborhood watch” (Factor II) were 0.90, 0.84, and 0.88 in the GEN sample, and 0.87, 0.79, and 0.84 in the CVOL sample, respectively. Correlations between all the CSES versions and the GCS-R, and the CSES and the BSCS indicated conceptual consistency. The CSES was positively correlated with the GCS-R in both the GEN and CVOL samples (*r* = ≥0.80). The CSES was also positively correlated with the BSCS in both samples (*r* = ≥0.64) (Table 4) (Additional files 1 and 2).

**Table 4:** CSES internal consistency and criteria-related validity

		GEN *N* = 3,484	CVOL *N* = 859
Basic statistics			
All CSES: 8 items	Mean (SD)	10.2 (5.2)	13.8 (4.6)
	Range	0–24	0–24
	Skewness	0.01	0.07
	Kurtosis	0.15	0.22
Community network: 4 items	Mean (SD)	5.7 (2.7)	6.3 (2.5)
	Range	0–12	0–12
	Skewness	0.04	0.07
	Kurtosis	0.23	0.17
Neighborhood watch: 4 items	Mean (SD)	5.4 (2.7)	7.8 (2.4)
	Range	0–12	1–12
	Skewness	0.09	0.20
	Kurtosis	0.26	0.44
Internal consistency			
All CSES: 8 items	Cronbach’s α	0.90	0.87
Community network: 4 items	Cronbach’s α	0.84	0.79
Neighborhood watch: 4 items	Cronbach’s α	0.88	0.84
Criterion-related validity			
GCS-R	R^a^	0.80***	0.86***
BSCS	R^a^	0.64***	0.66***

^a^Pearson’s correlation, ***p < 0.001

## Discussion

Local governments and community health service providers have implemented an array of interventions as social isolation and solitary death increases as a public health issue in developed countries. Many programs have targeted older people themselves [13–18], but not members of their communities. Preventing social isolation depends not only on older people themselves, but also on the members of the communities in which they live. Social isolation could be prevented by “mimamori” by a community, but a scale to measure the strength of such a community has not been addressed to date. To the best of our knowledge, our CSES is the first community-level measurement for preventing social isolation among older people.

The results support the adequate reliability, validity and useful structure of the CSES for evaluating the self-efficacy needed to prevent social isolation among older people, both in our GEN and CVOL groups. The CFA model verified factor validity and factor correctness of a set of eight observed variables within two factors: community network and neighborhood watch. Reliability was high (Cronbach’s alpha of all CSES versions was ≥0.87; community network was ≥0.87; and neighborhood watch was ≥0.80). The criterion-related validities were ≥0.80 (*p* < 0.001) between the scores of the CSES and GCS-R. Based on this collective evidence, the CSES demonstrated adequate psychometric properties to measure self-efficacy for preventing social isolation among older people, in both the GEN and CVOL groups. The results also demonstrated that the CSES could discriminate between the groups; that is, the scores of all the CSES factors and sub-scales in the CVOL group were higher than those in the GEN group (Table 4). This finding is also consistent with outcomes that volunteers have advantages over other citizens in terms of physical activity [42], mental health [43], self-rated health [44], and general self-efficacy [31].

There are similarities and differences between the CSES and other relevant concept or measures used in this field, e.g. self-esteem, self-confidence, and general self-efficacy. All of these concept or measures highlight the nature of beliefs about one’s self that are associated with self-value and ability. The measures differ, however, in the specificity of core construct and potential. The core construct of the CSES focuses on people’s beliefs in their own abilities to prevent social isolation among older people, in contrast to more generalized measures. Bandura [45] argued, “measures of self-percept must be tailored to the domain of psychological functioning being explored” (p. 396). Maibach and Murphy [46] also pointed out that measures of generalized self-efficacy have little explanatory or predictive value, whereas domain-related measures have considerable predictive value. Therefore, other measures have limited potential in targeting social isolation. The CSES, however, measures the community’s specific perceptions on addressing social isolation in older people. It strongly correlates with key aspects of actual performance to prevent social isolation among older people, predicting the community’s capacity and highlighting its potential to activate social capital in times of need.

How the tool will be used in public health setting? Measurements of Community’s Self-Efficacy in the CSES may not only grasp certain aspects of community or social context that affect outcomes in older people, but also inform community-level strategies and policies aimed at promoting public well-being in aging societies. That is, the CSES has potential utility for evaluating strengths and weaknesses of communities and promoting community development to help prevent social isolation among older people. For example, people with a heightened sense of the community’s self-efficacy can see preventing social isolation among older people as a challenging community health problem to be resolved, rather than a threat to be avoided, and also set challenging goals and demonstrate a stronger role for practice of *mimamori*. Conversely, people who have a low sense of community’s self-efficacy avoid difficult tasks and view them as a personal threat. They may have a weaker commitment to their goals and believe that difficult tasks and situations are beyond their abilities. Community’s self-efficacy change communities. Therefore, an effective program and system need to be developed to popularize the CSES for preventing social isolation among older people in communities and promoting public well-being in aging societies in the future.

The present study does have a few limitations. First, the design is cross-sectional. Although the literature and theory support the predictive ability of self-efficacy [47], the present study design does not allow for a causal determination between the CSES and achievement behavior as a final goal. Therefore, a prospective design is needed for determining the scale’s predictive validity. Second, although two major cities in Japan were used as the study settings, it would be useful to examine data in other community and/or country contexts. As Bandura [30] suggested, self-efficacy is not context-free, but rather is highly dependent on situational environment and community characteristics or social context.

Despite these limitations, this study still has important potential implications for public health. High levels of self-efficacy enhance one’s accomplishments and feeling of personal well-being [30]. Therefore *mimamori* can create a “win-win” community – older people benefit from the community people and the community people benefit from the act of ‘mimamori’ by establishing relationships of mutual trust. Community-based interventions for creating an atmosphere of friendliness and approachability, and facilitating daily interactions with neighbors, is worth considering as a public health strategy for encouraging help-seeking among older people who tend to be socially isolated [48] and community people respectively. If people can enjoy supportive relationships and thus prevent the trend toward social isolation, and if this can spread to rebuilding of relationships across society, older people and entire communities will be able to lead healthier and more agreeable and productive lives. We hope that such relationship building will not only lead to greater enjoyment of life, but also to improved social capital and health of the community as a whole.

## Conclusion

The CSES, which assesses community’s self-efficacy with regard to the notions of community networks and neighborhood watch, is a novel instrument with good psychometric properties for assessing self-efficacy for the purpose of community members preventing social isolation among older people. The CSES can contribute to understanding a community’s beliefs and help individuals at risk of social isolation, which can lead to solitary death. The CSES has potential utility for promoting health policies, practices, and interventions to promote well-being both in older people and the community as a whole.

## Additional files

Additional file 1: Appendix 1: English version of the final CSES. (PDF 226 kb)
Additional file 2: Appendix 2: Japanese version of the final CSES. (PDF 254 kb)

## Abbreviations

AGFI: Adjusted goodness-of-fit index
BSCS: Brief Sense of Community Scale
CFA: Confirmatory factor analysis
CFI: Comparative fit index
CSES: Community’s Self-Efficacy Scale for preventing social isolation among older people
CVOL: Community volunteer
GCS-R: The revised Generative Concern Scale
GEN: General population
GFI: Goodness-of-fit index
RMSEA: Root-mean-square error of approximation
