# Supplementary material for: Development of a community’s self-efficacy scale for preventing social isolation among community-dwelling older people (Mimamori Scale)
Source: BMC Public Health. 2016 Nov 28;16:1198. doi: 10.1186/s12889-016-3857-4 (PMC5127097; doi:10.1186/s12889-016-3857-4)
Supplement: Additional file 1: — Appendix 1: English version of the final CSES. (PDF 226 kb) [file 12889_2016_3857_MOESM1_ESM.pdf]

Appendix 1

Community's Self-Efficacy Scale for Preventing Social Isolation among Community-dwelling Older People (CSES; *Mimamori* Scale)

		Not confident at all	Slightly unconfident	Slightly confident	Completely confident
**Community Network (Total Possible Score: 0–12)**					
Q1	I can participate in the activities or volunteer work of my neighborhood association.	**0**	**1**	**2**	**3**
Q2	I can create an environment where my neighbors can comfortably gather.	**0**	**1**	**2**	**3**
Q3	I can encourage nearby neighbors to come out to gatherings.	**0**	**1**	**2**	**3**
Q4	I can discuss my concerns about residents at neighborhood gatherings or community meetings held by local government.	**0**	**1**	**2**	**3**
**Neighborhood Watch (Total Possible Score: 0–12)**					
Q5	I can check on older neighbors if I do not see them for a few days.	**0**	**1**	**2**	**3**
Q6	I can help older neighbors with grocery shopping, garbage disposal, and other chores.	**0**	**1**	**2**	**3**
Q7	I can check on neighborhood households where there are no signs of activity there.	**0**	**1**	**2**	**3**
Q8	When I notice a person I do not know in the neighborhood, I can speak to them.	**0**	**1**	**2**	**3**
**(Total Possible Scores: 0–24)**					

Tadaka E., et al.:Development of a Community's Self-Efficacy Scale for Preventing Social Isolation among Community-dwelling Older People (*Mimamori* Scale), BMC Public Health. 2016
