# Supplementary material for: Development of a community’s self-efficacy scale for preventing social isolation among community-dwelling older people (Mimamori Scale)
Source: BMC Public Health. 2016 Nov 28;16:1198. doi: 10.1186/s12889-016-3857-4 (PMC5127097; doi:10.1186/s12889-016-3857-4)
Supplement: Additional file 2: — Appendix 2: Japanese version of the final CSES. (PDF 254 kb) [file 12889_2016_3857_MOESM2_ESM.pdf]

Appendix 2

Community's Self-Efficacy Scale for Preventing Social Isolation among Community-dwelling Older People (CSES, *Mimamori* Scale)　（地域見守り自己効力感尺度日本語版）

	全く<br>自信がない	あまり<br>自信がない	やや<br>自信がある	大変<br>自信がある
**'コミュニティネットワーク'（0-12 点）**				
Q1　近所の人が気楽に集まる場をつくることができる。	**0**	**1**	**2**	**3**
Q2　周囲の住民に近所の集まりにでるように誘うことができる。	**0**	**1**	**2**	**3**
Q3　町内会（自治会）の活動、奉仕活動などに参加することができる。	**0**	**1**	**2**	**3**
Q4　気になる住民のことを町内会（自治会）や役所等の会議で話し合うことができる。	**0**	**1**	**2**	**3**
**'近隣見守り'（0-12 点）**				
Q5　隣近所の高齢者の顔を二、三日見ないときは声をかけることができる。	**0**	**1**	**2**	**3**
Q6　隣近所の高齢者の買い物やごみ出しなどを手助けすることができる。	**0**	**1**	**2**	**3**
Q7　物音や人の気配が全くしない隣近所の家は様子を確認することができる。	**0**	**1**	**2**	**3**
Q8　隣近所で見知らぬ人を見かけたら、一声かけることができる。	**0**	**1**	**2**	**3**
**合計（0-24 点）**				

Tadaka E., et al.:Development of a Community's Self-Efficacy Scale for Preventing Social Isolation among Community-dwelling Older People (*Mimamori* Scale), BMC Public Health. 2016
